# Supplementary material for: Silicon-based all-solid-state batteries operating free from external pressure
Source: Nat Commun. 2025 Jan 25;16:1013. doi: 10.1038/s41467-025-56366-z (PMC11761052; doi:10.1038/s41467-025-56366-z)
Supplement: Supplementary file 1 — Supplementary Information [file 41467_2025_56366_MOESM1_ESM.pdf]

Supplementary Information

Silicon-based all-solid-state batteries operating free from external pressure

Zhiyong Zhang[1], Xiuli Zhang[2], Yan Liu[3], Chaofei Lan[1], Xiang Han[4], Shanpeng Pei[1,5], Linshan Luo[1], Pengfei Su[1], Ziqi Zhang[6], Jingjing Liu[7], Zhengliang Gong[8], Cheng Li[8], Guangyang Lin[1], Cheng Li[1], Wei Huang[1], Ming-Sheng Wang[1,2]*, and Songyan Chen[1]**

[1] Department of Physics, Collaborative Innovation Center for Optoelectronic Semiconductors and Efficient Devices, Key Laboratory of Low Dimensional Condensed Matter Physics (Department of Education of Fujian Province), Jiujiang Research Institute, Xiamen University, Xiamen 361005, China

[2] State Key Lab of Physical Chemistry of Solid Surfaces, College of Materials, Xiamen University, Xiamen 361005, China

[3] School of Semiconductor Science and Technology, South China Normal University, Foshan 528225, China

[4] College of Materials Science and Engineering, Co-Innovation Center of Efficient Processing and Utilization of Forest Resources, Nanjing Forestry University, Nanjing 210037, China

[5] Shandong Electric Power Engineering Consulting Institute Corporation, Jinan 250031, China

[6] Science and Technology on Analog Integrated Circuit Laboratory, Chongqing 400000, China

[7] Microsoft Corporation, One Microsoft Way, Redmond, WA 98052

[8] College of Energy, Xiamen University, Xiamen 361102, China

Corresponding author: sychen@xmu.edu.cn, mswang@xmu.edu.cn, hanxiang@njfu.edu.cn

Keywords: all solid-state batteries, $Li_{21}Si_5$ alloy, silicon anode, pressure-free, ultra-high ICE

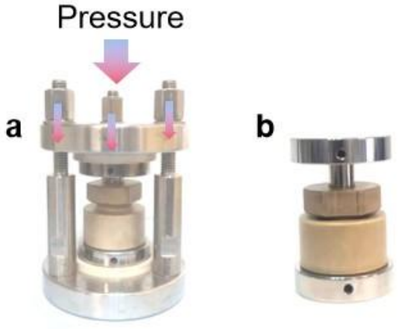

Supplementary Figure 1. (a) ASSBs operating high external pressure. (b) ASSBs operating free from external pressure.

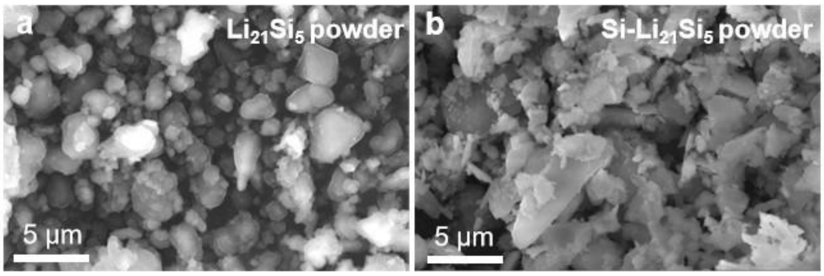

Supplementary Figure 2. SEM images of $Li_{21}Si_5$ and Si-$Li_{21}Si_5$ powder.

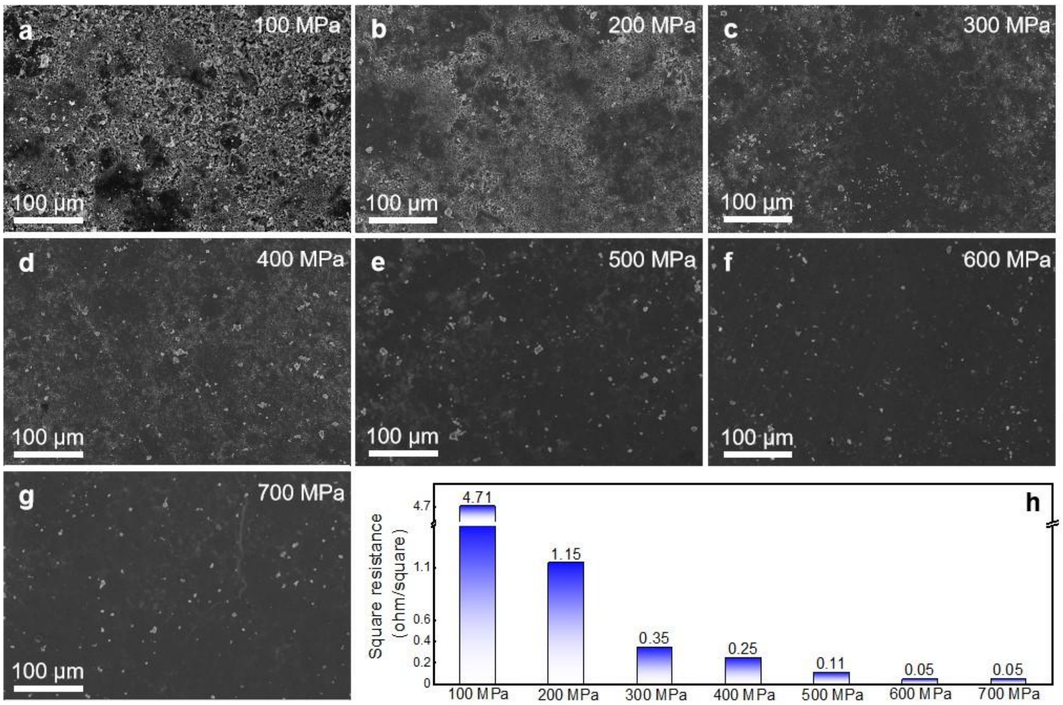

Supplementary Figure 3. SEM images of $Li_{21}Si_5$ powders cold-pressed at different stack pressure: (a) 100 Mpa, (b) 200 MPa, (c) 300 MPa, (d) 400 MPa, (e) 500 MPa, (f) 600 MPa, (g) 700 MPa. (h) Sheet resistance of cold-pressed $Li_{21}Si_5$ powders via different pressure.

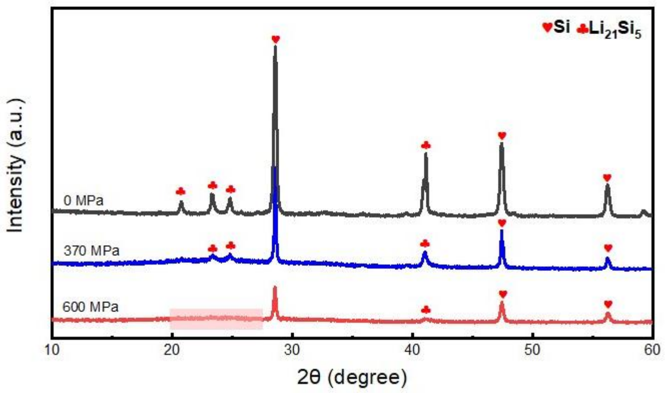

Supplementary Figure 4. XRD patterns of the Si-$Li_{21}Si_5$ powders sintered at a stack pressure of 600 MPa.

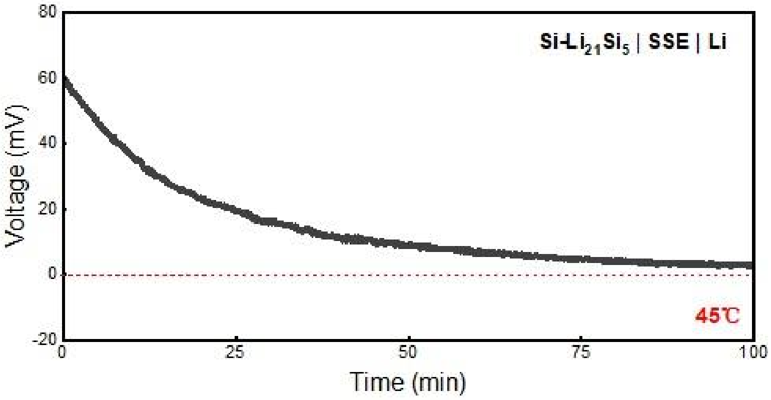

Supplementary Figure 5. Voltage profile during self-discharge. The test was performed without external pressure.

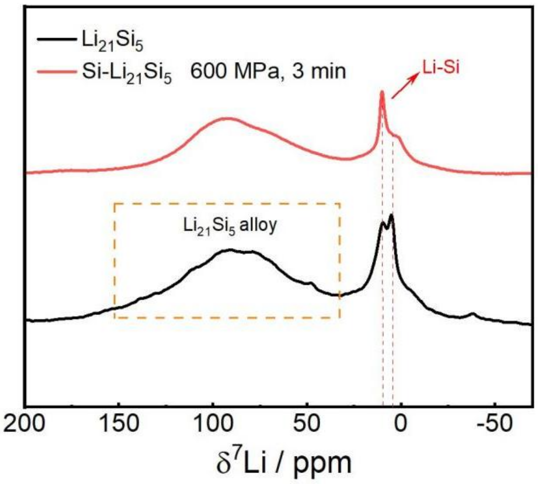

Supplementary Figure 6. NMR spectra of $Li_{21}Si_5$ powder and Si-$Li_{21}Si_5$ powders.

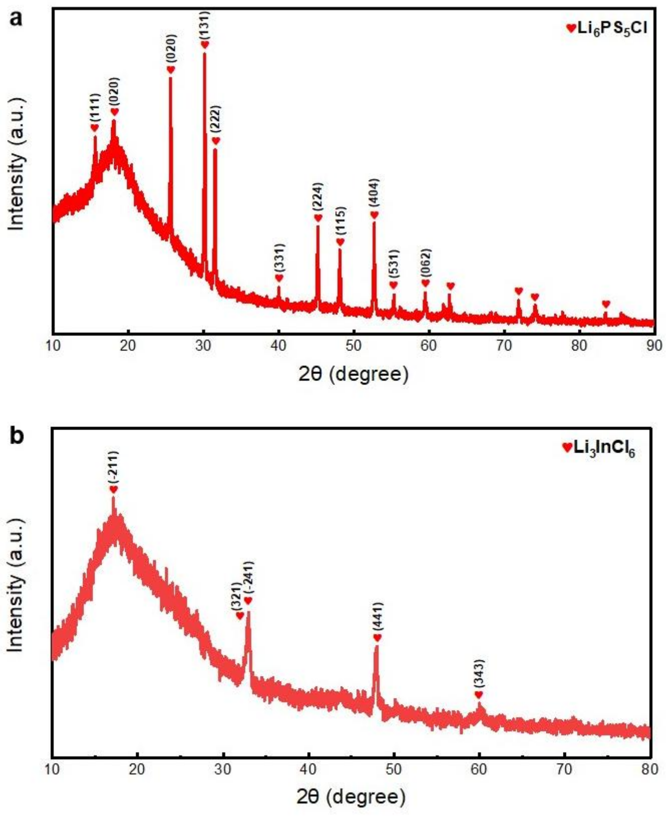

Supplementary Figure 7. XRD patterns of the Li_6PS_5Cl (a) and Li_3InCl_6 (b) powders.

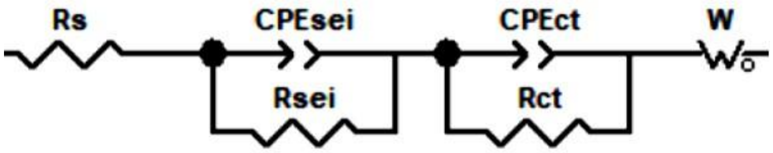

	Rs (ohm)	error% (Rs)	Rsei (ohm)	error% (Rsei)	Rct (ohm)	error% (Rct)
Si|SSE|LCO cell	55.3	0.58%	29.7	2.04%	201.8	0.69%
Si-$Li_{21}Si_5$|SSE|LCO cell	35.6	0.54%	12.6	5.96%	74.1	3.06%
$Li_{21}Si_5$/Si-$Li_{21}Si_5$|SSE|LCO cell	12.9	0.15%	5.3	0.70%	13.3	0.39%

Supplementary Figure 8. Corresponding equivalent circuit of EIS.

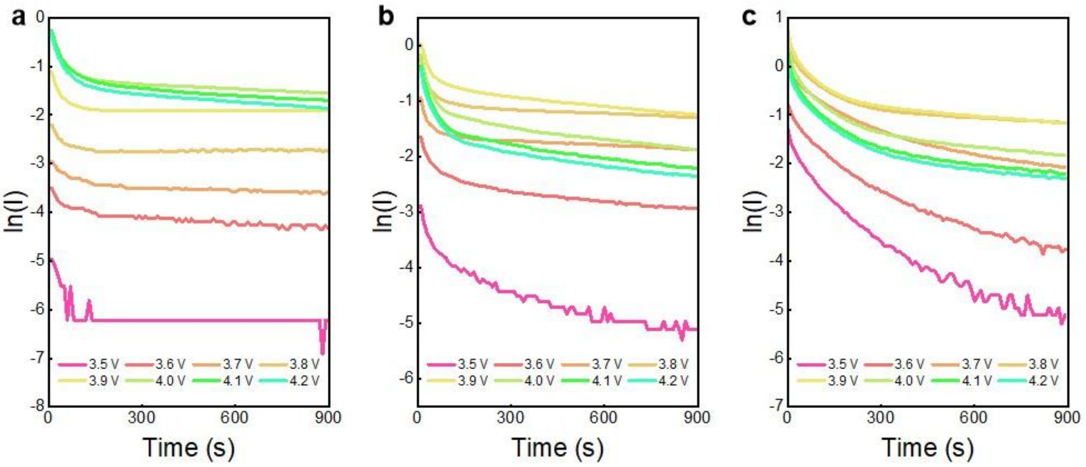

Supplementary Figure 9. PITT curves of Si|SSE|LCO (a), Si-$Li_{21}Si_5$|SSE|LCO (b), and $Li_{21}Si_5$/Si-$Li_{21}Si_5$|SSE|LCO (c). The PITT tests were operated at 45 ℃.

Supplementary Table 1 | Comparison of diffusion coefficient in this work and different anode in published literature.

References	Anode	Diffusion coefficients ($cm^2\ s^{-1}$)	Years
1	RGR	2.0×10^{-13}	2024
2	$Cr_{0.5}Nb_{24.5}O_{62}$	2.19×10^{-13}	2017
3	$W_3Nb_{14}O_{44}$	8.02×10^{-13}	2019
4	$Li_{0.38}Pr_{0.54}TiO_3$	1.80×10^{-12}	2024
5	$NiNb_2O_6$	1.20×10^{-12}	2022
6	$Si@Si_3N_4@C$	8.11×10^{-11}	2020
7	$Li_4Ti_5O_{12}$	3.89×10^{-11}	2022
8	$LiCrTiO_4$	1.54×10^{-11}	2017
9	$Li_{1.2}Ni_{2.5}B_2$	10^{-10}-10^{-11}	2024
10	LiMg	1.60×10^{-10}	2022
11	Co_2VO_4	3.14×10^{-10}	2022
12	$MnO_2@PNC$	10^{-8}	2022
13	Li_2MSiO_4	$10^{-6.5}$-$10^{-7.5}$	2022
14	LiC_6	2.0×10^{-7}	2024
This work	$Li_{21}Si_5$	$\geq2.09\times10^{-6}$	2024

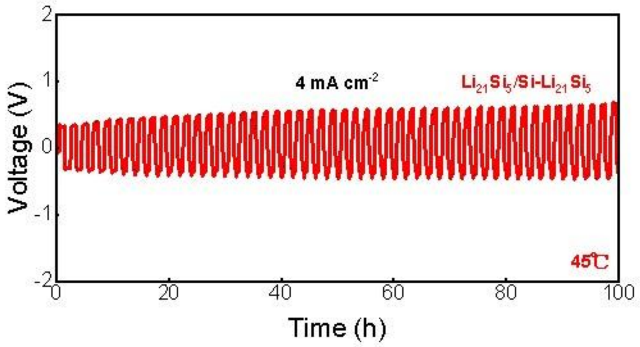

Supplementary Figure 10. Galvanostatic cycling of symmetric cell ($Li_{21}Si_5$/Si-$Li_{21}Si_5$|SSE|$Li_{21}Si_5$) at 4 mA cm^{-2}.

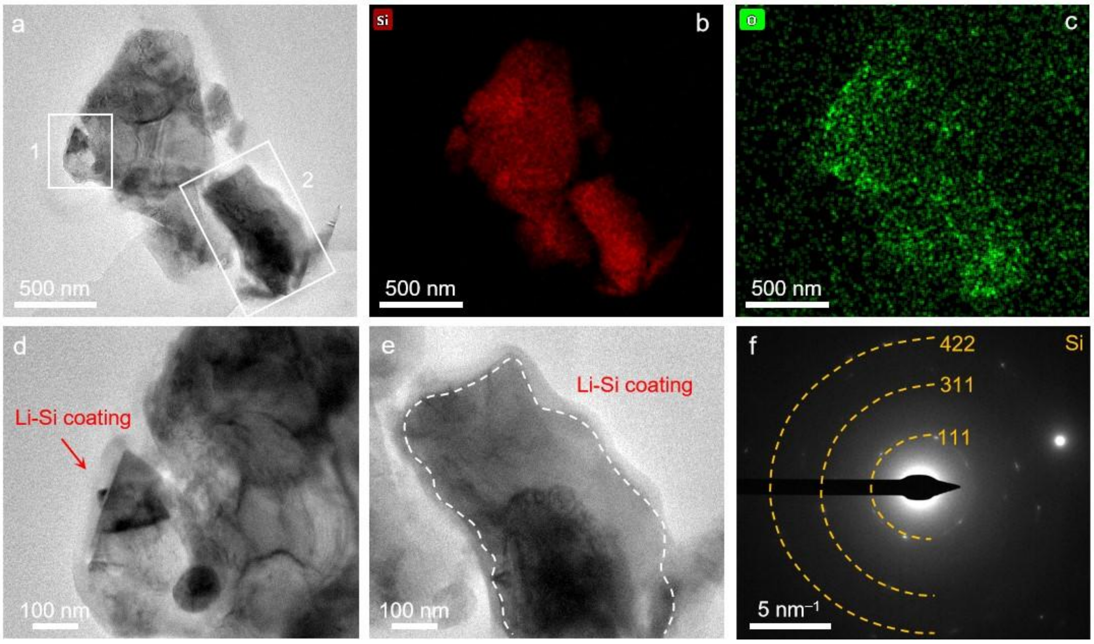

Supplementary Figure 11. (a) TEM image of Li-Si@Si particles. (b-c) Elemental mapping of Li-Si@Si particles. (d-e) HRTEM images of regions 1 and 2 in Supplementary Fig. 11a. (f) Selected area electron diffraction patterns of Li-Si@Si particles.

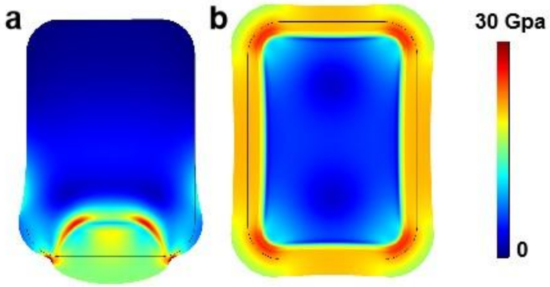

Supplementary Figure 12. Simulated volume expansion of Si particle (a) and Li-Si@Si particle (b) during lithiation.

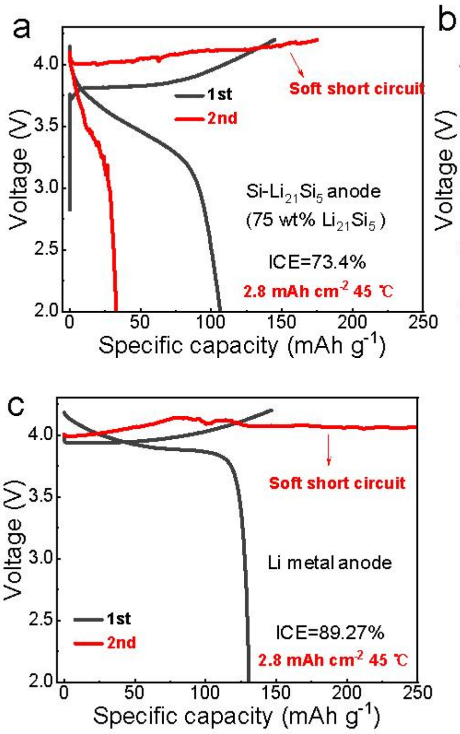

Supplementary Figure 13. Charge-discharge profiles of ASSBs at 2.3 mA cm^{-2} with Si-$Li_{21}Si_5$ anode ($Li_{21}Si_5$ mass ratio of 75%) (a), pure $Li_{21}Si_5$ anode (b), and Li metal anode (c).

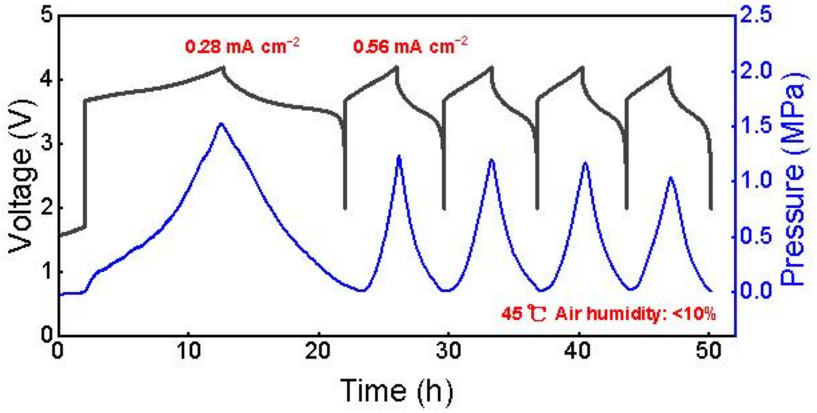

Supplementary Figure 14. Real-time pressure monitoring for $Li_{21}Si_5$/Si-$Li_{21}Si_5$-ASSB. Testing conditions: 45 °C, air atmosphere, air humidity: <10%.

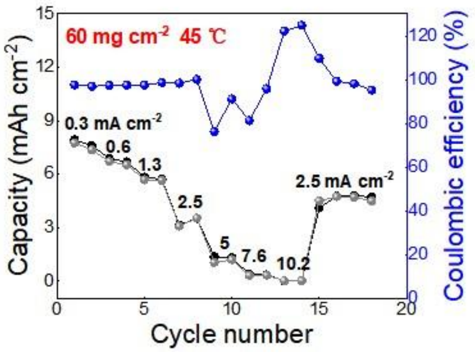

Supplementary Figure 15. Rate capability of $Li_{21}Si_5$/Si-$Li_{21}Si_5$-ASSBs with cathode mass loadings of 60 mg cm^{-2}.

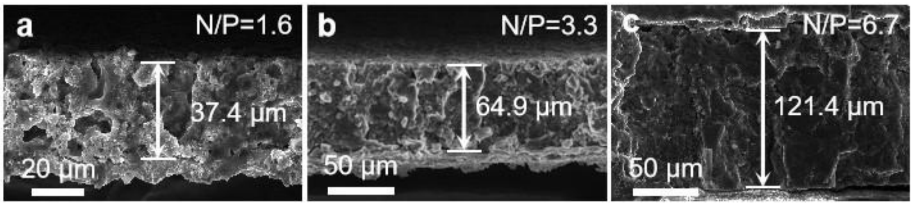

Supplementary Figure 16. Cross-section SEM images of $Li_{21}Si_5$/Si-$Li_{21}Si_5$ anode with N/P ratios of 1.6 (a), 3.3 (b), 6.7 (c) after cycling.

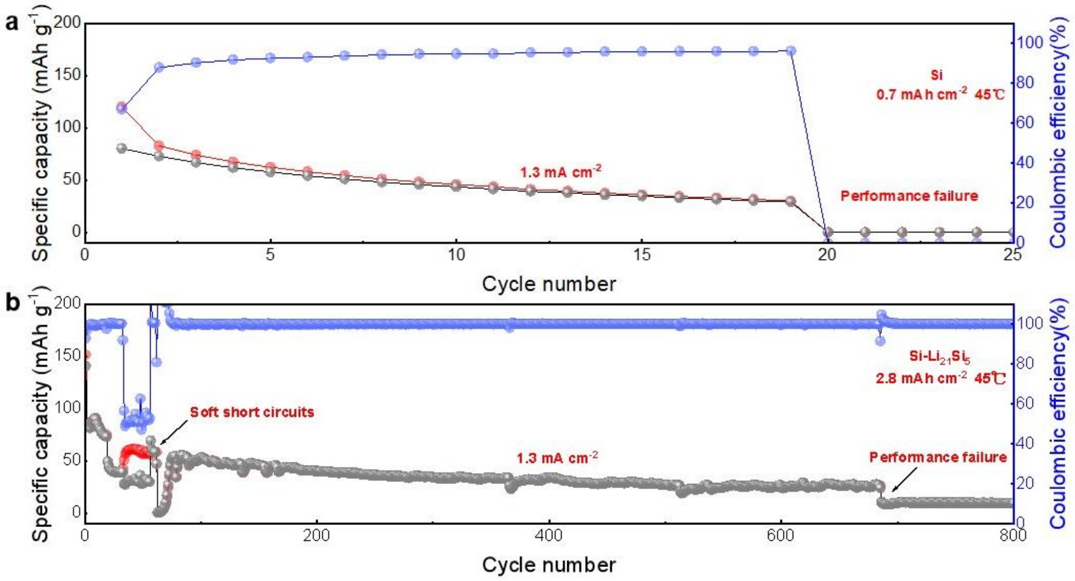

Supplementary Figure 17. Cycling performance of Si-ASSB and Si-$Li_{21}Si_5$-ASSB.

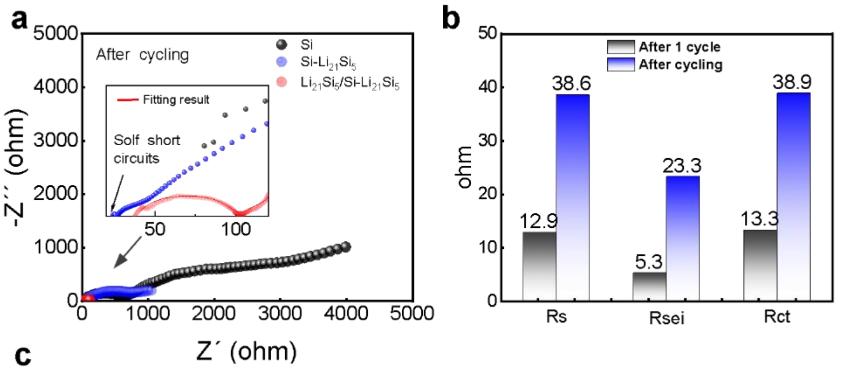

c

	Rs (ohm)	error% (Rs)	Rsei (ohm)	error% (Rsei)	Rct (ohm)	error% (Rct)
After 1 cycle	12.9	0.15%	5.3	0.70%	13.3	0.39%
After cycling	38.6	0.39%	23.3	1.67%	38.9	0.50%

Supplementary Figure 18. (a), EIS test of ASSBs using Si, Si-$Li_{21}Si_5$, and $Li_{21}Si_5$/Si-$Li_{21}Si_5$ anode after cycling. (b-c), Corresponding equivalent circuit of $Li_{21}Si_5$/Si-$Li_{21}Si_5$-ASSB after 1cycle and 1000 cycles.

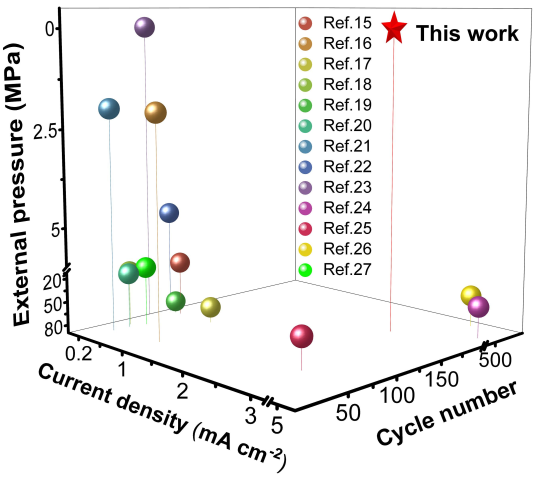

Supplementary Figure 19. Cycling performance of Si-ASSB and Si-$Li_{21}Si_5$-ASSB.

Supplementary Table 2 | Comparison of key parameters in this work and published literature.

Ref.	External pressure (Mpa)	Cycle number	Current density (mA cm^{-2})	Areal capacity (mAh cm^{-2})	Tempera-ture (°C)	Anode	Cathode
15	20	100	0.24	1	25	Si	NCM-111
16	2	35	1	3	80	Li	NMC
17	70	100	0.785	2	60	Si	NCA
18	20	50	0.2	2.7	25	Si	NCM
19	75	100	0.15	0.15	30	Li-In	NCM
20	20	45	0.28	1	30	Si	LCO
21	2	30	0.27	2.7	25	MXene/Mg	NCM-811
22	5	100	0.05	0.5	25	Li	NCA
23	0	80	0.008	0.05	25	Si	LFP
24	50	500	5	2	25	Si	NCM-811
25	50	62	2.8	0.5	25	Si	NCM
26	50	600	3	3.2	55	Si	LCO
27	15	60	0.32	1.6	30	Si	NCM
This work	0	183	2.5	2.8	45	Si	LCO

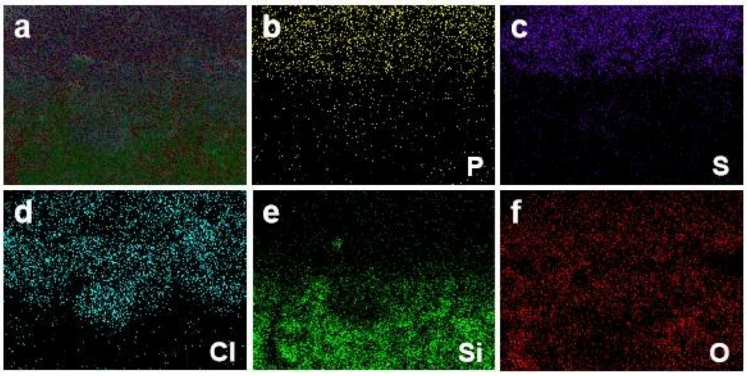

Supplementary Figure 20. EDS mappings of $Li_{21}Si_5$/Si-$Li_{21}Si_5$ anodes. The corresponding secondary electron SEM image is shown in Fig. 6e.

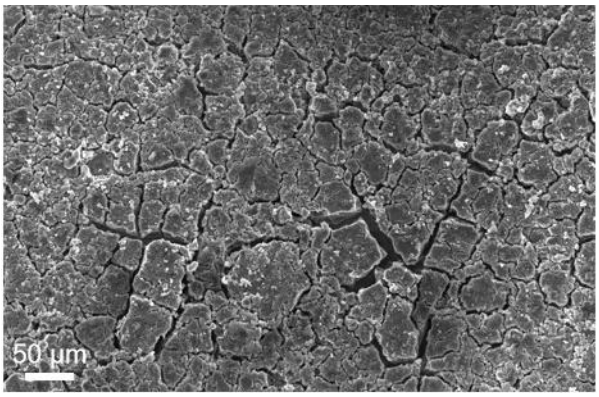

Supplementary Figure 21. SEM image of the surface of $Li_{21}Si_5$-ASSB after cycling.

Supplementary References

1. Chen, W. et al. Upcycling spent graphite into fast-charging anode materials through interface regulation. *ACS Energy Lett.* **9**, 3505-3515 (2024).
2. Yang, C. et al. $Cr_{0.5}Nb_{24.5}O_{62}$ nanowires with high electronic conductivity for high-rate and long-life lithium-ion storage. *ACS nano* **11**, 4217-4224 (2017).
3. Yan, L. et al. $W_3Nb_{14}O_{44}$ nanowires: ultrastable lithium storage anode materials for advanced rechargeable batteries. *Energy Storage Mater.* **16**, 535-544 (2019).
4. Liu, H. et al. A-site deficient perovskite lithium praseodymium titanate as a high-rate anode for lithium-ion batteries. *Chem. Eng. J.* **479**, 147765 (2024).
5. Xia, R. et al. Boukamp, B.; Kaghazchi, P., Nickel niobate anodes for high rate lithium-ion batteries. *Adv. Energy Mater.* **12***,* 2102972 (2022).
6. Xiao, Z. et al. Si@ Si_3N_4@C composite with egg-like structure as high-performance anode material for lithium ion batteries. *Energy Storage Mater.* **24**, 565-573 (2020).
7. Jin, X. et al. Mesoporous single-crystal lithium titanate enabling fast-charging Li-Ion batteries. *Adv. Mater.* **34**, 2109356 (2022).
8. Li, X. et al. Al doping effects on $LiCrTiO_4$ as an anode for lithium-ion batteries. *Rsc Adv.* **7**, 4791-4797 (2017).
9. Liu, W. et al. Ternary lithium nickel boride with 1D rapid-ion-diffusion channels as an anode for use in lithium-ion batteries. *Small* **20**, 2309918 (2024).
10. Siniscalchi, M. et al. On the relative importance of Li bulk diffusivity and interface morphology in determining the stripped capacity of metallic anodes in solid-state batteries. *ACS Energy Lett.* **7**, 3593-3599 (2022).
11. Ren, J. et al. Porous Co_2VO_4 nanodisk as a high-energy and fast-charging anode for lithium-ion batteries. *Nano-Micro Lett.* **14**, 1-14 (2022).
12. Yuan, X. et al. Mesoporous nitrogen-doped carbon MnO_2 multichannel nanotubes with high performance for Li-ion batteries. *Nano Energy,* **97**, 107235 (2022).
13. Zhang, J. et al. Fe saponite, a layered silicate for reversible lithium-ions storage with large diffusion coefficient. *J. Energy Chem,* **67**, 92-100 (2022).
14. Li, X. et al. Enabling fast mass transport in anode by a smartly built-in LiC_6 phase for high-performance solid-state lithium metal batteries. *Adv. Funct. Mater.* **34**, 2408447 (2024).
15. Han, S. Y. et al. Stress evolution during cycling of alloy-anode solid-state batteries. *Joule* **5**, 2450–2465 (2021).
16. Gao, X. et al. Solid-state lithium battery cathodes operating at low pressures. *Joule* **6**, 636–646 (2022).
17. Huang, Y., Shao, B., Wang, Y. & Han, F. Solid-state silicon anode with extremely high initial coulombic efficiency. *Energy Environ. Sci.* **16**, 1569–1580 (2023).
18. Cangaz, S. et al. Enabling high-energy solid-state batteries with stable anode interphase by the use of columnar silicon anodes. *Adv. Energy Mater.* **10**, 2001320 (2020).
19. Yamamoto, M., Terauchi, Y., Sakuda, A., Kato, A. & Takahashi, M. Effects of volume variations under different compressive pressures on the performance and microstructure of all-solid-state batteries. *J. Power Sources* **473**, 228595 (2020).

20. Kim, D. H. et al. Sheet-type Li_6PS_5Cl-infiltrated Si anodes fabricated by solution process for all-solid-state lithium-ion batteries. *J. Power Sources* **426**, 143–150 (2019).
21. Oh, J. et al. Anode-less all-solid-state batteries operating at room temperature and low pressure. *Adv. Energy Mater.* **13**, 2301508 (2023).
22. Doux, J. et al. Stack pressure considerations for room-temperature all-solid-state lithium metal batteries. *Adv. Energy Mater.* **10**, 1903253 (2020).
23. Chen, C. et al. Sustainable interfaces between Si anodes and garnet electrolytes for room-temperature solid-state batteries. *ACS Appl. Mater. Interfaces* **10**, 2185–2190 (2018).
24. Tan, D. H. S. et al. Carbon-free high-loading silicon anodes enabled by sulfide solid electrolytes. *Science* **373**, 1494–1499 (2021).
25. Xu, X. et al. Nano silicon anode without electrolyte adding for sulfide-based all-solid-state lithium-ion batteries. *Small* **19**, 2302934 (2023).
26. Zhang, Z. et al. An all-electrochem-active silicon anode enabled by spontaneous Li–Si alloying for ultra-high performance solid-state batteries. *Energy Environ. Sci.* **17**, 1061–1072 (2024).
27. Jun, S. et al. Interlayer engineering and prelithiation: empowering Si anodes for low-pressure-operating all-solid-state batteries. Small, 2309437 (2024).
