# Supplementary material for: Silicon-based all-solid-state batteries operating free from external pressure
Source: Nat Commun. 2025 Jan 25;16:1013. doi: 10.1038/s41467-025-56366-z (PMC11761052; doi:10.1038/s41467-025-56366-z)
Supplement: Supplementary file 2 — Description of Additional Supplementary Files [file 41467_2025_56366_MOESM2_ESM.docx]

**Supplementary Movie 1**

In-situ TEM observation of Si particle during lithiation.

**Supplementary Movie 2**

In-situ TEM observation of Li-Si@Si particle during lithiation.
