# Supplementary material for: Silicon-based all-solid-state batteries operating free from external pressure
Source: Nat Commun. 2025 Jan 25;16:1013. doi: 10.1038/s41467-025-56366-z (PMC11761052; doi:10.1038/s41467-025-56366-z)
Supplement: Supplementary file 5 — Reporting Summary [file 41467_2025_56366_MOESM5_ESM.pdf]

nature portfolio

Corresponding author(s): Songyan Chen
Last updated by author(s): Dec 19, 2024

Reporting Summary

Nature Portfolio wishes to improve the reproducibility of the work that we publish. This form provides structure for consistency and transparency in reporting. For further information on Nature Portfolio policies, see our Editorial Policies and the Editorial Policy Checklist.

Statistics

For all statistical analyses, confirm that the following items are present in the figure legend, table legend, main text, or Methods section.

n/a	Confirmed	
☐	☒	The exact sample size (n) for each experimental group/condition, given as a discrete number and unit of measurement
☐	☒	A statement on whether measurements were taken from distinct samples or whether the same sample was measured repeatedly
☒	☐	The statistical test(s) used AND whether they are one- or two-sided<br>*Only common tests should be described solely by name; describe more complex techniques in the Methods section.*
☒	☐	A description of all covariates tested
☒	☐	A description of any assumptions or corrections, such as tests of normality and adjustment for multiple comparisons
☐	☒	A full description of the statistical parameters including central tendency (e.g. means) or other basic estimates (e.g. regression coefficient) AND variation (e.g. standard deviation) or associated estimates of uncertainty (e.g. confidence intervals)
☒	☐	For null hypothesis testing, the test statistic (e.g. F, t, r) with confidence intervals, effect sizes, degrees of freedom and P value noted<br>*Give P values as exact values whenever suitable.*
☒	☐	For Bayesian analysis, information on the choice of priors and Markov chain Monte Carlo settings
☒	☐	For hierarchical and complex designs, identification of the appropriate level for tests and full reporting of outcomes
☒	☐	Estimates of effect sizes (e.g. Cohen's d, Pearson's r), indicating how they were calculated

Our web collection on statistics for biologists contains articles on many of the points above.

Software and code

Policy information about availability of computer code

Data collection	The electrochemical performance of ASSBs was measured by the CHI660E electrochemical workstation and LAND (CT2001A) battery tester.
Data analysis	The morphology and corresponding element distribution were characterized by field-emission scanning electron microscopy (FESEM, SIGMA-HD) and an energy dispersive X-ray detector, respectively. All the HRTEM experiments were conducted on a FEI Talos F200s operating at 200 kV. AFM images and area potential profile were simultaneously collected using the Kelvin probe force microscopy (KPFM, Bruker NW4). The in-situ TEM experiments were carried out using a FEI Talos F200s TEM equipped. The image scan rate was set at 0.3 s per line with a resolution of 128×128 pixels. The phase structure was detected by X-ray diffraction (XRD, Rigaku Ultima IV) with a Cu Kα source. 7Li solid-state nuclear magnetic resonance (SSNMR) were conducted on a Bruker NEO-600WB NMR spectrometer. Samples were packed in 1.3 mm rotors and spun at a speed of 15 kHz. The X-ray photoelectron spectroscopy was performed on the X-ray photoelectron spectrometer (XPS, Thermo Scientific K-Alpha).

For manuscripts utilizing custom algorithms or software that are central to the research but not yet described in published literature, software must be made available to editors and reviewers. We strongly encourage code deposition in a community repository (e.g. GitHub). See the Nature Portfolio guidelines for submitting code & software for further information.

Data

Policy information about availability of data

All manuscripts must include a data availability statement. This statement should provide the following information, where applicable:

- Accession codes, unique identifiers, or web links for publicly available datasets
- A description of any restrictions on data availability
- For clinical datasets or third party data, please ensure that the statement adheres to our policy

Provide your data availability statement here.

Research involving human participants, their data, or biological material

Policy information about studies with human participants or human data. See also policy information about sex, gender (identity/presentation), and sexual orientation and race, ethnicity and racism.

Reporting on sex and gender	Does not apply
Reporting on race, ethnicity, or other socially relevant groupings	Does not apply
Population characteristics	Does not apply
Recruitment	Does not apply
Ethics oversight	Does not apply

Note that full information on the approval of the study protocol must also be provided in the manuscript.

Field-specific reporting

Please select the one below that is the best fit for your research. If you are not sure, read the appropriate sections before making your selection.

☐ Life sciences ☐ Behavioural & social sciences ☒ Ecological, evolutionary & environmental sciences

For a reference copy of the document with all sections, see nature.com/documents/nr-reporting-summary-flat.pdf

Ecological, evolutionary & environmental sciences study design

All studies must disclose on these points even when the disclosure is negative.

Study description	Silicon-based all-solid-state batteries (Si-ASSBs) offer ultra-high energy density and safety but face significant application challenges due to the requirement of high external pressure. In this study, a Li21Si5/Si-Li21Si5 double-layered anode is developed for Si-ASSBs operating free from external pressure. Under the cold-pressed sintering of Li21Si5 alloys, the anode forms a top layer (Li21Si5 layer) with mixed ionic/electronic conduction and a bottom layer (Si-Li21Si5 layer) containing a three-dimensional continuous conductive network. The resultant uniform electric field at the anode/SSE interface eliminates the need for high external pressure and simultaneously enables a twofold enhancement of the lithium-ion flux at the anode interface. Such an efficient ionic/electronic transport system also facilitates the uniform release of cycling expansion stresses from the Si particles and stabilizes bulk-phase and interfacial structure of anode. Consequently, the Li21Si5/Si-Li21Si5 anode exhibited an impressive critical current density (CCD) of 10 mA cm−2 at a capacity of 10 mAh cm−2. And the Li21Si5/Si-Li21Si5-ASSBs achieve an ultra-high initial Coulombic efficiency (ICE) of 97.69% with high areal capacity of 2.8 mAh cm−2, as well as a low expansion rate of 14.5% after 1000 cycles. This work provides a promising anode design and new insight into the working mechanism of Si-ASSBs operating without external pressure for practical purposes.
Research sample	Si anode, Li21Si5/Si anode, and Li21Si5/Si-Li21Si5 anode.
Sampling strategy	This study compares the electrochemical properties, expansion stresses, interfacial structures and electric field states of anodes with different Li21Si5 alloy contents and different structures. The cold-pressing sintering effect and the self-discharge effect of Li21Si5 were employed to devise a double-conductor ionic/electronic layer for the anode on the upper surface and a three-dimensional conductive network for the anode on the lower surface. The expansion stress of the anode was homogenized and the interface was stabilized under conditions of cycling without external pressure.
Data collection	Z.Z. Zhang and S. Chen conceived the concept. Z.Z. Zhang carried out the synthesis and performed materials characterizations and electrochemical measurements. Z.Z. Zhang, S. Chen, M.S. Wang, Y. Liu, X. Zhang, and C. Lan inspired the synthesis method. Z.Z. Zhang, C. Lan, Y. Liu, P. Su, L. luo, G. Lin, C. Li, Z.Q. Zhang, and W. Huang conducted SEM, XRD and XPS test. C. Lan and Z.Z. Zhang conducted COMSOL modeling. Z.Z. Zhang, Z.L. Gong, and C. Li conducted real-time pressure monitoring test. X. Zhang, M.S. Wang, and Z.Z. Zhang conducted HRTEM and in-situ TEM. Z.Z. Zhang, M.S. Wang, S. Chen, X. Han, and J. Liu co-wrote the paper. All authors participated in the analysis of the experimental results.

Timing and spatial scale	Date collection spanned from 2022 to 2024, commencing at the initial stage of electrochemical testing and concluding upon the completion of the testing process.
Data exclusions	No date were excluded.
Reproducibility	All experiments were conducted at least three times.
Randomization	This work did not involve quantitative that required randomization.
Blinding	There was no blinding in our research.

Did the study involve field work? ☐ Yes ☒ No

Reporting for specific materials, systems and methods

We require information from authors about some types of materials, experimental systems and methods used in many studies. Here, indicate whether each material, system or method listed is relevant to your study. If you are not sure if a list item applies to your research, read the appropriate section before selecting a response.

Materials & experimental systems

n/a	Involved in the study
☒	☐ Antibodies
☒	☐ Eukaryotic cell lines
☒	☐ Palaeontology and archaeology
☒	☐ Animals and other organisms
☒	☐ Clinical data
☒	☐ Dual use research of concern
☒	☐ Plants

Methods

n/a	Involved in the study
☒	☐ ChIP-seq
☒	☐ Flow cytometry
☒	☐ MRI-based neuroimaging

Plants

Seed stocks	*Report on the source of all seed stocks or other plant material used. If applicable, state the seed stock centre and catalogue number. If plant specimens were collected from the field, describe the collection location, date and sampling procedures.*
Novel plant genotypes	*Describe the methods by which all novel plant genotypes were produced. This includes those generated by transgenic approaches, gene editing, chemical/radiation-based mutagenesis and hybridization. For transgenic lines, describe the transformation method, the number of independent lines analyzed and the generation upon which experiments were performed. For gene-edited lines, describe the editor used, the endogenous sequence targeted for editing, the targeting guide RNA sequence (if applicable) and how the editor was applied.*
Authentication	*Describe any authentication procedures for each seed stock used or novel genotype generated. Describe any experiments used to assess the effect of a mutation and, where applicable, how potential secondary effects (e.g. second site T-DNA insertions, mosiacism, off-target gene editing) were examined.*
